# Periaxin gene variants are linked to age-related cataracts in Cx46 deficient lenses

**DOI:** 10.1038/s42003-025-08722-4

**Published:** 2025-09-24

**Authors:** Chun-hong Xia, Eddie Wang, Lin Li, Dong Wang, Bo Chang, Mei Li, Xiaohua Gong

**Affiliations:** 1 Vision Science and School of Optometry, University of California, Berkeley, Berkeley, CA USA; 2 The Jackson Laboratory, Bar Harbor, ME USA; 3 The UCB/UCSF jointed graduate program in Bioengineering, University of California, Berkeley, Berkeley, CA USA

**Keywords:** Genetic interaction, Lens diseases

## Abstract

Genetic predisposition affects cataract severity and progression, but no specific genetic modifier has been identified to date. This study reveals *Periaxin (Prx)* gene variants that cause four amino acid substitutions in the cytoskeletal scaffold protein Periaxin (PRX) between C57BL/6J (B6) and 129S4 (129) mouse strains, modulating the severity of age-related cataracts in connexin 46 knockout (Cx46KO) mice. Expression of 129-PRX is significantly higher than B6-PRX in the lens. Additionally, 129-PRX is broadly distributed across lens fibers, accumulates at fiber cell tricellular vertices, and co-localizes with actin filaments at surface protrusions in inner fibers and cultured cells. Aberrant membrane/F-actin aggregates and irregular fibers appear only in the 129-Cx46KO lens core with severe nuclear cataracts. These findings suggest that Cx46 deficiency and the gain-of-function 129-*Prx* variant synergistically disrupt fiber cell homeostasis and promote membrane/F-actin aggregation, leading to severe age-related cataracts.

## Abstract (web summary)

Periaxin variants and Cx46 knockout disrupt lens fiber cell homeostasis and cytoskeletal organization, contributing to the severity of age-related cataracts.

## Introduction

Cataracts, characterized by a clouding of the lens, remain the leading cause of blindness worldwide. The mechanisms underlying the progression and severity of cataracts in humans and animal models are not well understood. Currently, no non-surgical method effectively prevents, delays, or treats cataracts. The etiology of cataracts is influenced by multiple factors, including inherited genetic disorders, systemic diseases, environmental risk factors, and aging^1–5^. Cataract pathology, as well as the onset and progression of cataracts, are linked to a wide range of molecular and cellular alterations, including disruption of lens cellular and subcellular structures, decreased solubility and stability of lens proteins, and abnormal post-translational modifications^4,6^. The complex molecular and cellular mechanisms underlying the pathology of both hereditary and age-related cataracts remain poorly characterized, primarily due to the concurrent changes required for maintaining lens transparency and the onset of cataract formation. While predisposed genetic variances are believed to contribute to human age-related nuclear cataracts^7–9^, no specific genetic modifiers regulating cataract progression and severity have been identified to date^10^. In this study, we investigate potential genetic modifiers of cataracts, initially observed in connexin 46 (*Cx46* or *Gja3*) knockout (Cx46KO) mice across different strain backgrounds^11^, and successfully identify a genetic modifier that influences cataract severity.

The lens comprises densely packed elongated fiber cells, overlaid by a monolayer of epithelial cells on the anterior surface, all enclosed within the basement membrane known as the lens capsule^12^. Lens growth depends on the proliferation of epithelial cells, as well as the differentiation and elongation of newly formed fibers at the lens equator^13,14^. Lens fiber cells are elongated and hexagonally shaped, precisely stacked, and gradually develop surface interlocking structures, including ball-and-sockets on the broad sides and protrusions at the tricellular vertices, subsequently leading to the formation of interior mature fiber^15–18^. Lens fibers rely on surface junctions and interlocking structures, including protrusions and ball-and-sockets, to maintain mechanical stability during lens accommodation^19–21^. Fully elongated interior fibers undergo a maturation process within a narrow organelle-free zone, where all intracellular organelles are eliminated^22–24^. These maturing interior fibers transition through a remodeling zone, where their shape and surface interlocking structures undergo continuous reorganization, eventually forming mature fibers (MF) that are essential for maintaining the lens’s optical clarity and refractive power^25,26^. In the lens core, MF are tightly packed and exhibit undulating surface structures known as ridges and valleys^27–29^.

All lens fiber cells are connected by intercellular gap junction channels formed by Cx46 (alpha3 connexin) and Cx50 (alpha8 connexin), which are encoded by the *Gja3* and *Gja8* genes, respectively^30^. Cx46 is essential for electrical coupling between interior MF, as these fibers become uncoupled in Cx46KO lenses^31^. Mutations in either Cx46 or Cx50 are linked to various types of nuclear cataracts in both humans and mice^32–34^. Cx46KO mice develop postnatal, age-related nuclear cataracts^35,36^, which are associated with increased insolubility and degradation of crystallin proteins, elevated calcium levels, activation of calcium-dependent calpains, and calcification in Cx46 deficient lenses^35,37–39^. In contrast, Cx50 knockout (Cx50KO) mice have smaller lenses and mild nuclear cataracts^23,40^, with reduced electrical coupling among interior MF^41^. Interestingly, Cx50KI46 knockin (Cx46KI) lenses, in which the endogenous Cx50 is replaced by the knockin Cx46, remain transparent but are slightly smaller than age-matched wild-type lenses^42^. This suggests that Cx46KI prevents the development of mild nuclear cataracts caused by Cx50 deficiency. These studies demonstrate that Cx46 is critical for maintaining lens transparency by providing fiber-to-fiber communication in the lens core.

Unexpectedly, Cx46KO mice develop dense nuclear opacity only in the 129 strain genetic background (129-Cx46KO), including both the 129SvJae (129S4) and 129Svj (129S1) substrains, while displaying very mild nuclear opacity in the C57BL/6J strain genetic background (B6-Cx46KO)^11^. This observation suggests that genetic modifiers between the 129 and B6 strain genetic backgrounds significantly influence cataract formation in Cx46KO lenses. In contrast, Cx50KO mice exhibit mild nuclear cataracts that are minimally influenced by the 129 and B6 genetic backgrounds^43^, indicating a less pronounced strain-dependent effect. Thus, the Cx46KO mouse model offers a valuable system for identifying novel genetic modifiers of age-related nuclear cataracts by comparing the B6 and 129 strain genetic backgrounds. We propose that the B6 genetic background may contain cataract-suppressing factors, while the 129 genetic background may harbor cataract-promoting factors.

Studies of 129-Cx46KO mice have shown that calpain 3, a calcium-activated protease encoded by *Capn3*, is essential in mediating age-related nuclear cataract formation, as cataract severity is reduced in double-knockout (Cx46KO/Capn3KO) lenses^36^. Calpain 3 is known to be activated during fiber cell differentiation^44^. Several calpains are likely to play important roles in remodeling the membrane cytoskeleton during fiber cell maturation and cataract formation^45^. However, lens gene expression studies and proteomic analyses reveal no significant differences in calpain 3 expression levels between the 129 and B6 mouse strains, and no missense mutations in the *Capn3* gene have been detected^46,47^. These findings suggest that calpain 3 is not a genetic modifier influencing cataract severity in Cx46KO lenses. Instead, its activation may be regulated by unidentified genetic modifiers, making calpain 3 a valuable biomarker for further investigation. To date, the specific genetic suppressors of age-related nuclear cataracts in Cx46KO mice have yet to be identified^11^.

Through genome-wide mapping and targeted analysis of selective chromosomal crossover mice, we identified four missense variants in the *Periaxin* (*Prx*) gene on chromosome 7 (Chr7) that differ between the 129 and B6 strain backgrounds. Periaxin (PRX), a scaffold protein, plays a critical role in regulating fiber cell shape and morphogenesis in the lens^48^. Variations in the *Prx* gene significantly impact both the expression levels and spatial distribution of PRX proteins in lens fiber cells, thereby affecting their function. This study reveals a novel molecular genetic mechanism in which Cx46 deficiency-induced disruptions in lens calcium homeostasis, combined with *Prx* variant-driven defects in fiber cell morphogenesis, synergistically enhance the severity of age-related nuclear cataracts.

## Results

### Mapping periaxin gene variants to the genomic interval modulating cataract severity in Cx46KO mice across B6 and 129 strain backgrounds

**Fig. 1:**
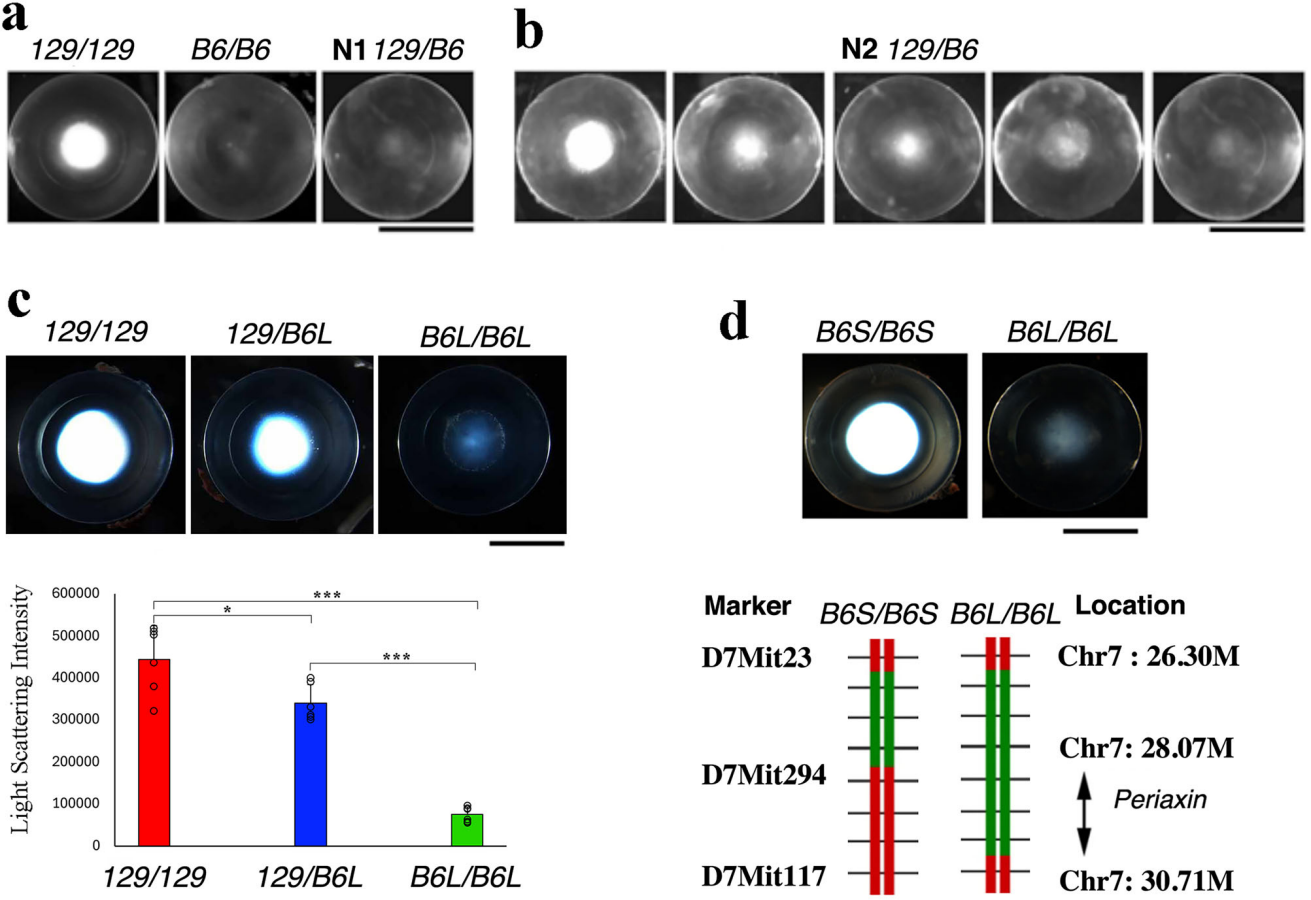
A cataract suppressor is mapped to a chromosome 7 interval where the periaxin gene is located. **a** Lens images of *Cx46KO* mice reveal a severe nuclear cataract in the 129-strain genetic background (*129/129*), a very mild cataract in the B6-strain genetic background (*B6/B6*), and a mild cataract in the first-generation mixed genetic background of 129 and B6 (N1 *129/B6*) at 4 weeks of age. Scale bar, 1 mm. **b** Cx46KO lens images from second-generation littermates with mixed genetic backgrounds (N2 *129/B6*) at 4 weeks old, generated by intercrossing the N1 129/B6 Cx46KO mice, show variable nuclear cataracts ranging from mild to intermediate to severe opacities. Scale bar, 1 mm. **c** Lens images of 6-week-old N14 littermates show a severe nuclear cataract in a 129 genetic background mouse (*129/129*), an intermediate cataract in a heterozygous *129/B6L* mouse (one Chr7-*129* allele and one Chr7-*B6L* allele), and a mild cataract in a homozygous *B6L/B6L* mouse. These littermates were generated by intercrossing N13 heterozygous Chr7(*129/B6L*) Cx46KO male and female mice. A bar graph compares the light scattering intensity of 6-week-old Cx46KO lenses across the *129/129*, *129/B6L*, and *B6L/B6L* backgrounds (*P < 0.05, ***P < 0.001; *n* = 6 lenses per genotype). Scale bar, 1 mm. **d** Representative lens images of 6-week-old Cx46KO mice from two different Chr7 crossover lines: *B6S/B6S* and *B6L/B6L*. The *B6S/B6S* mouse displays a severe cataract, while the *B6L/B6L* mouse exhibits a mild cataract. The lower panel illustrates the Chr7 mapping results for these two crossover lines using the markers D7Mit23, D7Mit294, and D7Mit117. The *B6L/B6L*-*Cx46KO* mouse line carries two copies of a long Chr7-B6L fragment (~4.4 Mb of B6 genomic DNA flanked by markers D7Mit23 and D7Mit117, shown in green), while the *B6S/B6S*-*Cx46KO* line contains two copies of a short Chr7-B6 fragment (<2 Mb of B6 genomic DNA located between D7Mit23 and D7Mit294, shown in green). The 129 Chr7 genomic DNAs are shown in red. The cataract suppressor is mapped to the 28.07–30.71 Mb interval between the linkage markers D7Mit294 and D7Mit117, where the *periaxin* gene is located. Scale bar, 1 mm.

We performed a genome-wide linkage analysis to identify genetic modifiers linked to the severity of nuclear cataracts in Cx46KO mice on the 129 and B6 mouse strain genetic backgrounds^11^. Consistent with previous findings, Cx46KO mice developed severe age-related nuclear cataracts only in the 129 strain background (129/129), while mild cataracts were observed in the B6 strain background (B6/B6)^36^. We further observed that all mice in the first generation (N1) of the 129/B6 mixed strain background (N1 129/B6), produced by mating 129- and B6-Cx46KO mice, developed mild cataracts (Fig. 1a). The mild cataract phenotype observed in all N1 129/B6-Cx46KO mice suggests the presence of dominant cataract suppressor(s) in the B6 stain genetic background. The second-generation (N2) littermates, produced by intercrossing N1 129/B6-Cx46KO males and females, exhibited cataracts of varying severity, ranging from mild to severe (Fig. 1b). Out of 140 N2 129/B6-Cx46KO mice, 40 developed severe lens opacities, 39 exhibited intermediate opacities, and 61 showed mild opacities. The data from the N2 129/B6-Cx46KO mice suggest that multiple genetic modifiers contribute to the variation in cataract severity observed between the B6 and 129 strain genetic backgrounds.

To identify unknown genetic modifiers, we first conducted a genome-wide linkage analysis of quantitative trait loci (QTL). QTLs were determined by comparing genomic DNA samples from two cohorts of N2 Cx46KO mice: 20 with severe cataracts and 20 with mild cataracts. Linkage analysis revealed that markers D2Mit148, D7Mit230, and D9Mit11 had LOD scores of 5.724, 3.925, and 2.975, respectively, all exceeding the 99% threshold score of 2.419. This indicates a significant association between these loci and cataract severity. Genomic DNA samples from 200 mice with severe cataracts and 200 mice with mild cataracts were collected for further linkage mapping with additional markers; however, the results did not conclusively narrow the intervals at these three loci.

Based on our hypothesis that the B6 strain genetic background harbors dominant suppressors that alleviate the severe cataract phenotype, we predicted that some third-generation (N3) offspring–generated by backcrossing N2 129/B6-Cx46KO mice with mild cataract into 129*-*Cx46KO mice–would develop mild or intermediate cataracts, rather than the severe cataracts typically seen in 129-Cx46KO lenses. To test this hypothesis, we designed a breeding strategy in which N2 129/B6-Cx46KO mice with mild to intermediate cataracts were selectively backcrossed with 129-Cx46KO mice. The N3 offspring, aged 6–7 weeks, underwent direct visual examination of cataract severity using a slit-lamp. Some N3 129/B6-Cx46KO mice developed mild to intermediate cataracts. N3 mice with intermediate cataracts were further backcrossed with 129-Cx46KO mice to generate the fourth-generation (N4) offspring. Consistently, some N4 129/B6-Cx46KO mice also exhibited intermediate cataracts at 6–7 weeks of age. This finding supports our hypothesis that a dominant cataract suppressor in the B6 strain genetic background alleviates cataract severity in 129/B6-Cx46KO mice, leading to intermediate cataracts rather than severe cataracts, as typically observed in 129-Cx46KO mice. For each backcrossing generation, two to three 129/B6-Cx46KO mice exhibiting intermediate cataracts were bred with 129-Cx46KO mice to generate the next generation of 129/B6-Cx46KO offspring. With each successive generation, the proportion of the B6 strain genetic background was gradually reduced. Phenotypic screening was performed for each generation. Through phenotypic screening of backcrossed offspring, we identified and characterized 12th-generation (N12) 129/B6-Cx46KO mice that continued to exhibit intermediate cataracts. Using linkage analysis using markers on chromosomes 2, 7, and 9, we identified an N12 129/B6-Cx46KO male with intermediate cataracts. This mouse carried a ~4.4 Mb B6-Chr7 genomic crossover fragment, designated as the B6L allele, flanked by markers D7Mit23 and D7Mit117 on 129-Chr7. This N12 male 129/B6L-Cx46KO mouse was crossed with 129-Cx46KO females to generate 13^th^-generation (N13) 129/B6L-Cx46KO male and female offspring. More than 100 14th-generation (N14) offspring resulting from the intercross of N13 129/B6L-Cx46KO males and females were phenotypically and genotypically characterized. The results revealed that approximately 25% of offspring with the B6L/B6L*-*Chr7 genotype developed mild cataracts, 25% with the 129/129-Chr7 genotype exhibited severe cataracts, and about 50% with the 129/B6L-Chr7 genotype displayed mild to intermediate cataracts (Fig. 1c). These findings confirm the presence of a semi-dominant cataract suppressor within the B6L interval on chromosome 7.

To further refine the cataract suppressor locus within the B6L fragment, N13 129/B6L-Cx46KO mice were backcrossed with 129-Cx46KO mice, generating crossover offspring with progressively smaller B6 genomic intervals. Through this process, we identified a Cx46KO female mouse carrying a ~1.8 Mb B6-Chr7 fragment spanning 26.30–28.07 Mb, flanked by markers D7Mit23 and D7Mit294, which we designated as the B6S allele. We established two Cx46KO backcross mouse lines that were homozygous for either the B6L or B6S alleles, and confirmed that both lines carry the CP49 gene deletion characteristic of the 129 strain genetic background. B6L/B6L-Cx46KO mice developed mild cataracts, while B6S/B6S-Cx46KO mice exhibited severe cataracts, closely resembling those observed in 129-Cx46KO mice (Fig. 1d, upper panel). These findings suggest that the semi-dominant cataract suppressor is localized within the B6-Chr7 interval spanning 28.07–30.71 Mb, flanked by markers D7Mit294 and D7Mit117.

**Fig. 2:**
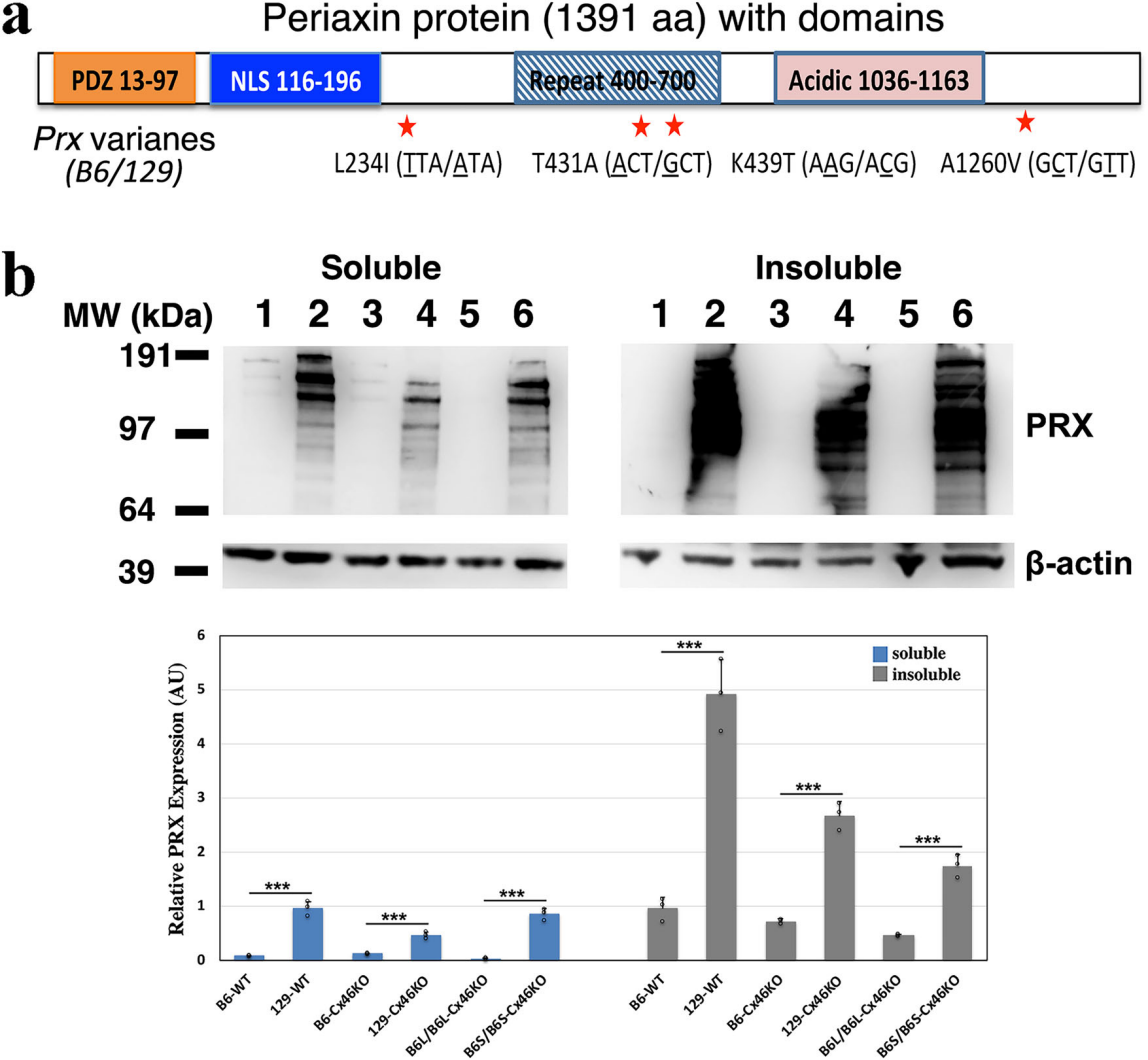
B6-Prx and 129-Prx genes contain four missense SNPs, showing low and high PRX protein levels in the lens, respectively. **a** A schematic map illustrates the structure of the periaxin protein, consisting of 1391 amino acid residues. The protein includes a PDZ domain at the N-terminal region, followed by a nuclear localization signal (NLS), a repeat domain, and an acidic region near the C-terminal. Four missense SNPs differentiate the B6 and 129 mouse strains, resulting in four amino acid residue substitutions: L234I, T431A, K439T, and A1260V, as indicated by red stars. **b** Western blot analysis of PRX protein expression was performed on total lens water-soluble and water-insoluble protein fractions from 3-week-old mouse lenses. The lanes were labeled as follows: (1) B6 wild-type (B6-WT), (2) 129 wild-type (129-WT), (3) B6-*Cx46KO*, (4) 129-*Cx46KO*, (5) *B6L/B6L-Cx46KO*, and (6) *B6S/B6S-Cx46KO*. A distinct cluster of PRX protein bands, ranging between 97 and 192 kDa molecular weight markers, were prominently detected in the 129 lens samples (*129-WT*, lane 2; and *129-Cx46KO*, lane 4*)* and in the *B6S/B6S-Cx46KO* lens sample (lane 6). In contrast, PRX proteins were barely detectable in B6 lens samples (*B6-WT*, lane 1; and *B6-Cx46KO*, lane 3) as well as in the *B6L/B6L*-*Cx46KO* lens sample (lane 5). In the bar graph shown below, protein band intensities were quantified and normalized to the β-actin band. Data are presented as mean ± SD (*n* = 3), with statistical analysis performed using Student’s *t*-test (***P < 0.001). Both 129-WT and 129-Cx46KO samples exhibited significantly higher PRX protein levels in both water-soluble and water-insoluble fractions compared to their B6-strain counterparts (B6-WT and B6-Cx46KO). Furthermore, B6S/B6S-Cx46KO lenses exhibited higher levels of 129-PRX protein, whereas B6L/B6L-Cx46KO lenses displayed lower levels of B6-PRX protein.

We conducted a search for nonsynonymous coding candidate genes within the genomic interval between D7Mit294 and D7Mit117 using the mouse genome SNP database (http://www.informatics.jax.org/snp). This search identified eight genes–*Prx*, *Map3K10*, *Zfp780b*, *Gm4636*, *Il28a*, *Rasgrp4*, *Fam98c*, and *Zfp30–*that contain nonsense SNP variants leading to amino acid residue substitutions in their encoded proteins. To evaluate their potential relevance to lens function, we examined the functions of these genes and their expression levels in the lens, using data from the literature, the NCBI expression database, and transcriptomic datasets from a previously published study^49^. Among these, *Prx* emerged as the only potential modifier within the B6L interval (Fig. 1d, lower panel), supported by studies on PRX protein function^50^ and lens pathology observed in *Prx* knockout mice^48^. No compelling evidence in the literature supports the involvement of the other seven candidate genes in lens function. PRX plays a critical role in regulating fiber cell shape and size, fiber-fiber radial alignment, and lens stiffness^48,51^. Sequencing data from the GeneBank database reveals four missense SNPs in the coding region of the *Prx* gene that distinguish the B6 and 129 mouse strain genetic backgrounds. These four SNPs result in amino acid substitutions (L234I, T431A, K439T, and A1260V) between the B6-PRX and 129-PRX proteins (Fig. 2a). These findings suggest that PRX proteins, encoded by variants of the *Prx* gene, act as genetic modifiers that influence cataract severity in Cx46KO lenses across the 129 and B6 mouse strains.

### Distinct expression of 129-PRX and B6-PRX in lens fibers: high and widespread distribution of 129-PRX versus low and restricted distribution of B6-PRX

PRX was initially identified as a cytoskeletal scaffold component within the Ezrin-Periaxin-Periplakin-Desmoyokin (EPPD) complex in lens fibers^50^. It plays a crucial role in maintaining lens fiber cell shape, as *Prx* knockout lenses exhibit disorganized and deformed fibers, although cataract formation is not observed^48^. To investigate the potential mechanisms by which 129-PRX and B6-PRX proteins influence cataract severity in Cx46KO lenses, we subcloned the *Prx* coding regions from RNA samples isolated from wild-type and Cx46KO mice with 129, B6, and B6L genetic backgrounds. Sequencing of B6-Prx and 129-Prx cDNAs confirmed the presence of four missense variants, consistent with those listed in the GenBank database (Fig. 2a). Western blot analysis revealed significantly higher 129-PRX protein levels in both the water-soluble and water-insoluble fractions of lens samples from mice carrying the 129*-Prx* alleles, including 129 wild-type (129-WT), 129-Cx46KO and B6S/B6S*-*Cx46KO (Fig. 2b, samples 2, 4, and 6). In contrast, B6-PRX protein levels were low in the water-soluble fractions and barely detectable in the water-insoluble fractions of lens samples from mice carrying the B6*-Prx* alleles, including B6-WT, B6-Cx46KO, and B6L/B6L-Cx46KO (Fig. 2b, samples 1, 3, and 5). β-actin protein levels in both water-soluble and water-insoluble fractions were comparable across all lens samples. Densitometric analysis of the Western blot bands revealed that 129-PRX protein levels were significantly higher than B6-PRX in both the water-soluble and water-insoluble fractions, showing a 5- to 10-fold increase in 129 lens samples compared to equivalent B6 lens samples from either wild-type or Cx46KO mice (Fig. 2b). To investigate whether the genetic background of mouse strains influences *Prx* transcriptional regulation, quantitative real-time PCR (qRT-PCR) was performed to measure *Prx* transcript levels in wild-type and Cx46KO lenses from both 129 and B6 strains (Supplementary Fig. 1). The results showed that 129 lenses exhibited approximately a twofold increase in *Prx* transcript levels compared to their B6 counterparts (i.e., 129-WT vs B6-WT, and 129-Cx46KO vs B6-Cx46KO). These findings suggest that the elevated transcript level of 129-*Prx* may contribute–at least in part–to the markedly increased 129-PRX protein levels observed in the lens, in addition to the impact of the protein variant itself.

**Fig. 3:**
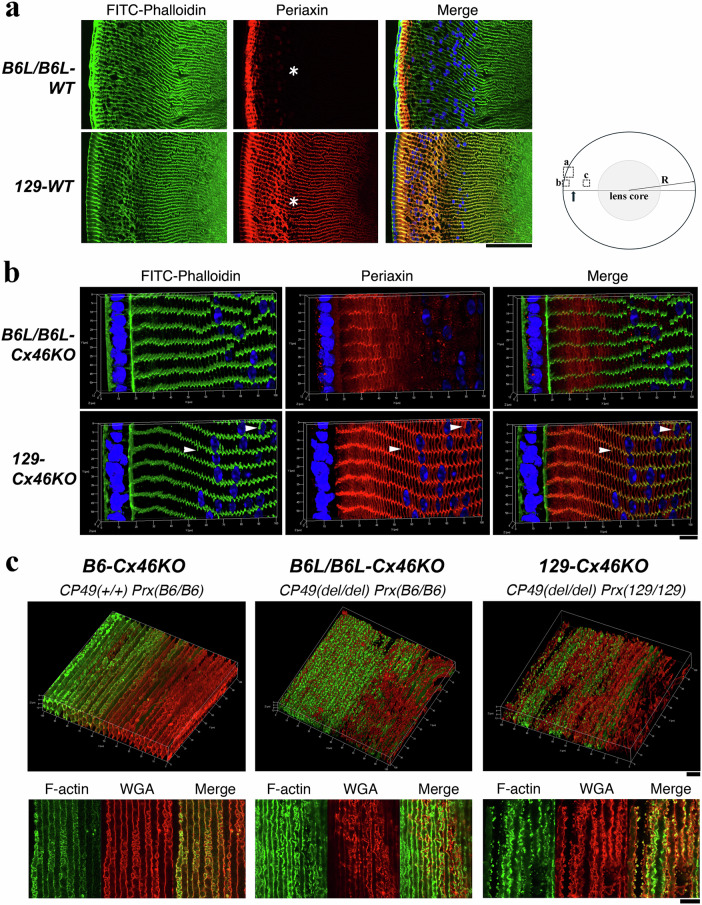
B6-PRX protein distribution is restricted to peripheral lens fibers, whereas 129-PRX proteins are broadly distributed in both peripheral and interior fibers. **a** Frozen cross-sections of 129 wild-type (*129-WT*) and B6L wild-type *(B6L/B6L-WT*) lenses from 3-week-old mice were immunostained with FITC-phalloidin to label F-actin (green), anti-periaxin antibody (red), and DAPI for nuclei (blue). B6-PRX proteins are exclusively detected in peripheral differentiating fiber cells, approximately 50 μm from the lens surface, while 129-PRX proteins are present in both peripheral and interior fibers. White asterisks indicate inner lens fibers. Scale bar: 50 μm. The schematic lens illustration shows the locations of the imaged areas in panels a, b and c. The arrow marks the denucleation zone, approximately 150 μm from the lens surface. R indicates the lens radius (~1 mm), and the shadowed area represents the cataract. **b** Three-dimensional (3D) imaging of lens vibratome cross-sections was performed with triple-labeling with FITC-phalloidin (green), anti-periaxin antibody (red), and DAPI (blue). In the *B6L/B6L-Cx46KO* lens, B6-PRX proteins are localized at the cell boundaries of peripheral differentiating fibers, approximately 50 μm from the lens surface. The staining signals become punctate in inner fibers and gradually diminish. In contrast, in the *129-Cx46KO* lens, 129-PRX proteins are detected at the cell boundaries of both peripheral and interior fibers, with pronounced enrichment at tricellular vertices. White arrowheads indicate elongated protrusions, positive for both F-actin and PRX, which are observed along the long sides of hexagonal-shaped fiber cells specifically in the 129-strain background. Lenses are isolated from 3-week-old mice. Scale bar: 10 µm. **c** 3D images of anterior-posterior (A/P) lens vibratome sections from 3-week-old B6-*Cx46KO*, 129-*Cx46KO*, and *B6L/B6L-Cx46KO* mice were stained with FITC-phalloidin and rhodamine-WGA and reconstructed from a 100 μm × 100 μm area. B6-*Cx46KO* mice carry wild-type CP49 (CP49(+/+)), whereas both 129-*Cx46KO* and *B6L/B6L-Cx46KO* contain a CP49 gene deletion (CP49(del/del)) from the 129 strain genetic background. The upper panels show 3D reconstructions of F-actin (green) and WGA (red) staining in inner mature fibers, located approximately 300–400 μm from the lens surface within the transitional region, where mature fibers on the right side (toward lens core) display reduced F-actin staining. The lower panels present corresponding 2D images of F-actin and WGA labeling from the same transitional regions shown in the upper panels. Scale bars: 10 μm.

To rule out any potential influence of strain genetic backgrounds alone on the distribution of 129-PRX and B6-PRX protein in the lens, we bred B6L/B6L-Cx46KO mice with 129-WT mice to generate the B6L/B6L-WT mouse line. This line shares the same genetic background as the 129 mouse strain, except for the ~4.4 Mb B6-Chr7 genomic fragment from the B6L allele. Next, we examined the distributions of B6-PRX and 129-PRX in lenses from mice with identical genetic backgrounds by comparing B6L/B6L-WT to 129-WT and B6L/B6L-Cx46KO to 129-Cx46KO. Immunohistological data revealed that lens fiber organization remained normal in the cortex of B6L/B6L-WT, 129-WT, B6L/B6L-Cx46KO, and 129-Cx46KO lenses (Fig. 3). The data further showed that B6-PRX proteins were predominantly localized to the most peripheral fiber cells, spanning approximately 20–30 cell layers, or around 50 μm in depth from the lens surface, in both B6L/B6L-WT lenses (Fig. 3a, upper panels) and B6L/B6L-Cx46KO lenses (Fig. 3b, upper panels). High-magnification images revealed that B6-PRX proteins were expressed at the cell boundaries of peripheral hexagonally shaped fibers, transitioning into intracellular punctate dots in inner fibers before disappearing (Fig. 3b, upper middle panel). In contrast, 129-PRX proteins were extensively distributed in both peripheral and interior fibers, reaching at least 200 μm in depth from the lens surface in both 129-WT (Fig. 3a, lower panels) and 129-Cx46KO (Fig. 3b, lower panels) lenses. Moreover, 129-PRX protein staining signals were significantly reduced in fibers deeper than 200 μm. High-magnification images revealed that 129-PRX proteins were expressed at all cell boundaries, with a high concentration at the tricellular vertices (Fig. 3b, lower panels). Additionally, 129-PRX was found in abnormally elongated F-actin-positive protrusions, extending deeply along the long sides of hexagonally shaped fiber cells in 129-Cx46KO lenses (indicated by white arrowheads in Fig. 3b, lower panels). In contrast, such elongated protrusions were not observed in B6L/B6L-Cx46KO lens fibers. These findings clearly demonstrate distinct differences in the distribution of B6-PRX and 129-PRX proteins in the lens cortical fibers of both wild-type and Cx46KO lenses.

PRX is a scaffold protein essential for maintaining the shape and alignment of interior fiber cells by regulating protein complexes within the cytoskeleton, membrane, and extracellular protein networks^48,51,52^. Since B6-PRX proteins were detected only in the peripheral fibers of B6-WT and B6-Cx46KO lenses (Fig. 3a, b), we hypothesized that the higher expression and broader distribution of 129-PRX may represent a gain-of-function variant, potentially disrupting the remodeling of deeper inner MF and contributing to cataract formation. To test this hypothesis, we obtained morphological data of inner mature fiber cells in deeper regions by double labeling with FITC-phalloidin to stain F-actin and rhodamine-WGA to stain cell membranes. High-resolution three-dimensional (3D) and two-dimensional (2D) confocal images revealed the distribution of membrane-associated F-actin and cell shape in the inner MF of B6-Cx46KO, B6L/B6L-Cx46KO, and 129-Cx46KO lenses (Fig. 3), which exhibited very mild, mild, and severe cataracts, respectively (Fig. 1). Noticeable changes in cell shape and membrane-associated F-actin distribution were observed in the transitional zone of deep inner MF, approximately 300–400 μm from the lens equatorial surface (Fig. 3c). In the B6-Cx46KO lens, inner MF maintained a typical hexagonal shape and remained well-aligned, with F-actin signals enriched at the tricellular junctions (Fig. 3c, left side of the upper left panel). However, as the fibers extended beyond approximately 350 μm in depth toward the lens core, membrane-associated F-actin signals were markedly diminished (Fig. 3c, right side of the upper left panel). A 2D sectional image of the B6-Cx46KO lens inner MF revealed distinct surface mushroom-shaped interlocking protrusions along the flat regions of mature fiber cell boundaries (Fig. 3c, bottom left panel). The 3D image of B6L/B6L-Cx46KO inner mature lens fibers revealed a similar distribution and reduction of F-actin signals as observed in B6-Cx46KO lens fibers (Fig. 3c, middle panels). However, it also displayed a mixture of irregular and hexagonal-shaped fibers, along with noticeable disruption in both WGA and F-actin signals at the fiber cell boundaries (Fig. 3c, upper middle panel). The 2D image showed irregular surface interlocking protrusions, with unevenly distributed WGA signals along the fiber cell boundaries (Fig. 3c, bottom middle panel). The 3D image of 129-Cx46KO inner mature lens fibers showed a complete loss of hexagonal fiber cell architecture due to severe fiber cell deformation, accompanied by a disrupted distribution of both F-actin and WGA signals (Fig. 3c, top right panel). The 2D WGA image further revealed irregularly shaped fibers with aberrantly distributed surface protrusions, some of which exhibited highly concentrated or accumulated F-actin signals (Fig. 3c, bottom right panel). These findings suggest that cataract severity is closely linked to the degree of F-actin signal disruption, fiber cell deformation, and the formation of abnormal surface interlocking structures in deep inner MF. This supports our hypothesis that the gain-of-function 129-PRX variant impairs membrane-cytoskeletal structures, including surface interlocking structures, thereby contributing to the severe cataract formation observed in the core of 129-*Cx46KO* lenses. To further support this hypothesis, we performed high-resolution imaging analysis to examine 129-PRX protein localization within the surface interlocking structures of individual fiber cells across both the peripheral and inner regions of 129-WT and 129-Cx46KO lenses.

### 129-PRX protein association with mature fiber surface interlocking structures, fiber deformation, and membrane/F-actin aggregates in the lens core of 129-Cx46KO mice

**Fig. 4:**
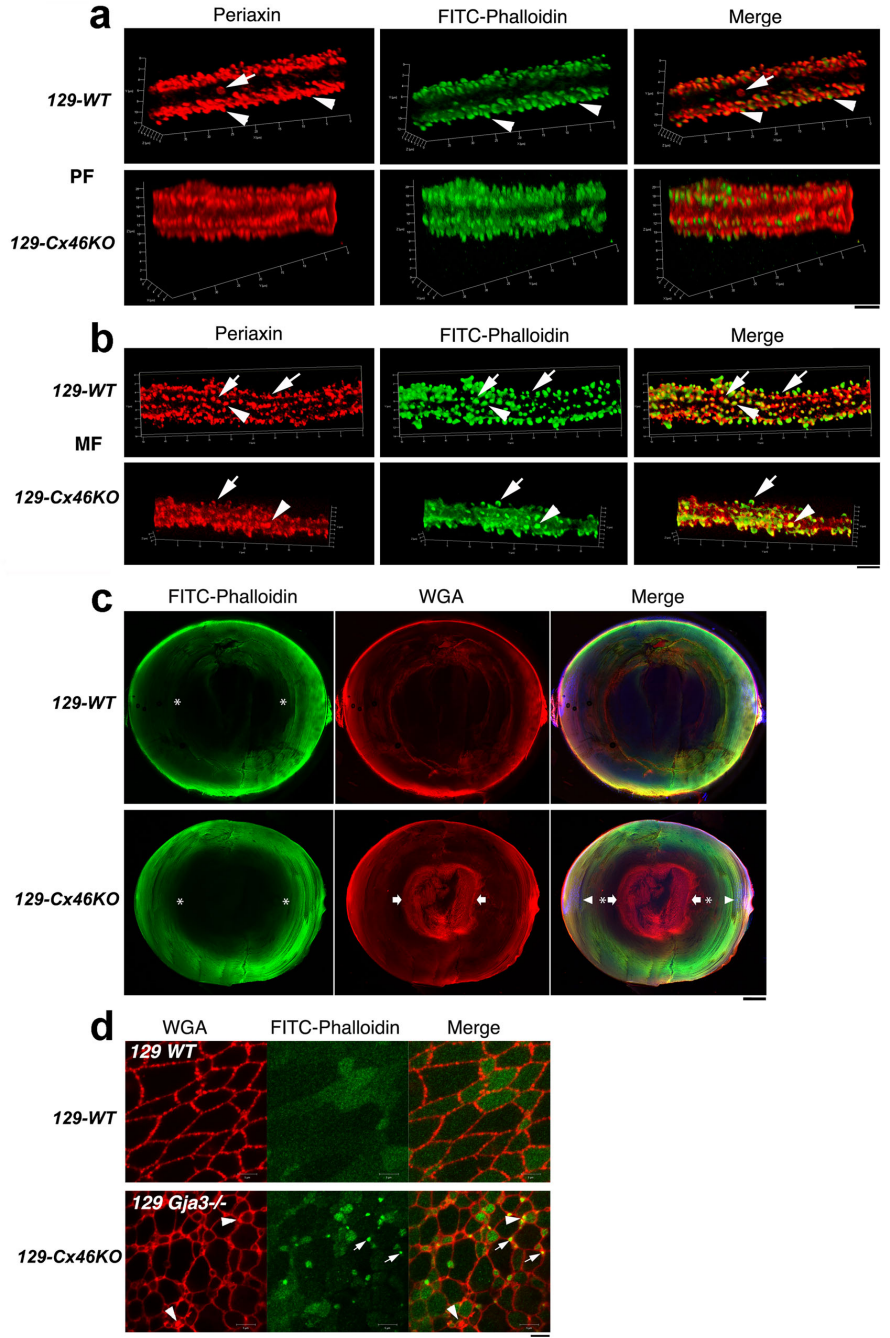
129-PRX proteins are associated with severe nuclear cataracts in *129-Cx46KO* mice. **a** 3D images of individual peripheral fiber (PF) immunostained with an anti-periaxin antibody (red) and FITC-phalloidin (green) from 3-week-old 129-WT and 129-Cx46KO lenses. White arrowheads indicate protrusions on the short sides, while white arrows mark the ball-and-sockets on the long side. Scale bar: 5 µm. **b** 3D images of individual inner maturing fiber (MF) immunostained with an anti-periaxin antibody (red) and FITC-phalloidin (green) from 3-week-old 129-WT and 129-Cx46KO lenses. White arrowheads indicate protrusions with colocalization of 129-PRX and F-actin, while white arrows mark protrusion with segregated distributions of 129-PRX and F-actin signals. Scale bar: 5 µm. **c** Deformed fibers and membrane aggregates in the 129-*Cx46KO* lens core. Low-magnification images of lens A/P sections from 3-week-old *129-WT* (upper panels) and *129-Cx46KO* mice (lower panels), triple-labeled with DAPI (blue), FITC-phalloidin (green), and WGA (red). DAPI staining reveals a denucleation zone at approximately 150 μm from the lens equatorial surface (white arrowheads). FITC-phalloidin staining shows an obvious reduction of F-actin in inner mature fibers, starting around 350 μm from the lens equatorial surface (white asterisks). Rhodamine-WGA labeling displays intense WGA signals in the *129-Cx46KO* lens core, starting in mature fibers around 500 μm from the lens equatorial surface (white arrows), correlating with the region of the nuclear cataract. Scale bar: 100 μm. **d** A comparison of lens core fibers from 3-week-old 129*-WT and 129-Cx46KO* mice, double labeled with Rhodamine-WGA and FITC-phalloidin. White arrowheads indicate WGA-labeled fiber cell membrane aggregates, while white arrows point to FITC-phalloidin-positive aggregates or regions in *129-Cx46KO lenses*. Scale bar: 5 μm.

Confocal immunostaining images of individual peripheral fibers (PF) and inner MF from both 129-WT and 129-Cx46KO lenses revealed a marked accumulation of 129-PRX proteins within fiber cell surface interlocking structures, including protrusions and ball-and-sockets (Fig. 4a, b). In the hexagonally shaped PF of the 129-WT lens, 3D images showed that 129-PRX and F-actin were highly enriched and predominantly colocalized at protrusions (white arrowheads) along the short sides and at tricellular junctions (Fig. 4a, upper panels). Additionally, 129-PRX proteins were also detected within ball-and-sockets along the long sides (white arrows) (Fig. 4a, upper panels). In the individual PF of the 129-Cx46KO lens, 3D images showed that both 129-PRX and F-actin were enriched at protrusions; however, the staining signals around these protrusions appeared noticeably diffuse, and deformation of the hexagonal shape of the fiber cells was evident (Fig. 4a, lower panels). In individual MF of the 129-WT lens, 129-PRX and F-actin signals were either colocalized or segregated along the aligned protrusions (Fig. 4b, upper panels). Some protrusions showed clear colocalization of 129-PRX and F-actin (Fig. 4b, upper panels, white arrowheads), while others displayed segregated distributions of 129-PRX and F-actin signals (Fig. 4b, upper panels, white arrows). In contrast, individual MF of the 129-Cx46KO lens exhibited severely deformed cell shapes, misaligned and irregular protrusions, with diffused or irregular staining signals of 129-PRX and F-actin on both the fiber cell bodies and the aberrant protrusions (Fig. 4b, lower panels). Despite these abnormalities, some 129-PRX and F-actin signals remained highly enriched at the abnormal protrusions, where the signals were either colocalized (Fig. 4b, lower panels, white arrowheads) or segregated (Fig. 4b, lower panels, white arrows). High-resolution single-fiber imaging datasets revealed that severely deformed inner MF, with disrupted protrusions and altered F-actin distributions, were observed only in 129-Cx46KO lenses, but not in 129-WT lenses. These findings suggest that 129-PRX plays a critical role in driving inner mature fiber deformation and the formation of abnormal protrusions, specifically in Cx46-deficient lenses.

To directly evaluate the relationship between fiber cell deformation and the early stages of nuclear cataract formation in the lens core, we performed confocal imaging analysis to examine the intensity and distribution of F-actin and WGA-positive membrane signals in whole lens sections from 3-week-old 129-WT and 129-Cx46KO mice, using triple-labeling with FITC-phalloidin, rhodamine-WGA, and DAPI. In 129-WT lenses, low-magnification images revealed that phalloidin-positive F-actin and WGA-positive membrane signals were stronger in the lens cortex and gradually diminished toward the lens core, where only weak signals were observed (Fig. 4c, upper panels). Similarly, low-magnification images of 129-Cx46KO lens sections showed a comparable F-actin distribution from the cortex to the lens core and a similar distribution of WGA-positive membrane signals in the cortex. However, the 129-Cx46KO lens core exhibited an unexpectedly intense accumulation of WGA-positive membrane signals (Fig. 4c, lower panels), corresponding to the region of severe nuclear cataract formation. High-magnification images revealed that in the 129-WT lens core, fiber cells maintained their typical polygonal shapes and uniform sizes in cross-sectional view (Fig. 4d, upper panels). These fibers exhibited evenly distributed WGA-positive signals along the plasma membrane, while some also showed weak and diffuse F-actin signals within their cytosol (Fig. 4d, upper panels). In contrast, fiber cells in the 129-Cx46KO lens core exhibited severely irregular shapes and highly variable sizes in cross-section (Fig. 4d, lower panels). Additionally, these fibers displayed WGA-positive membrane aggregates, along with numerous small fibers containing extensive F-actin-positive aggregates, which were either adjacent to or colocalized with the WGA-positive membrane aggregates (Fig. 4d, lower panels). These findings suggest that the early stages of nuclear cataract formation in the 129-Cx46KO lens core are characterized by extensively deformed or abnormally small core fibers containing F-actin and/or WGA-positive aggregates. Furthermore, they indicate that nuclear cataract may be initiated by disrupted membrane-cytoskeletal structures in the MF of the lens core due to the presence of 129-PRX in Cx46-deficient lenses. This fiber disruption leads to the accumulation of severely deformed fibers with extensive membrane/F-actin aggregates, contributing to nuclear cataract in the 129-Cx46KO lens core. To further investigate the molecular mechanisms underlying this process, we examined the interaction between 129-PRX and other proteins involved in regulating membrane/F-actin complexes in cultured lens cells in vitro.

### Colocalization of 129-PRX with F-actin and ezrin in differentiated lens fibers in vitro

**Fig. 5:**
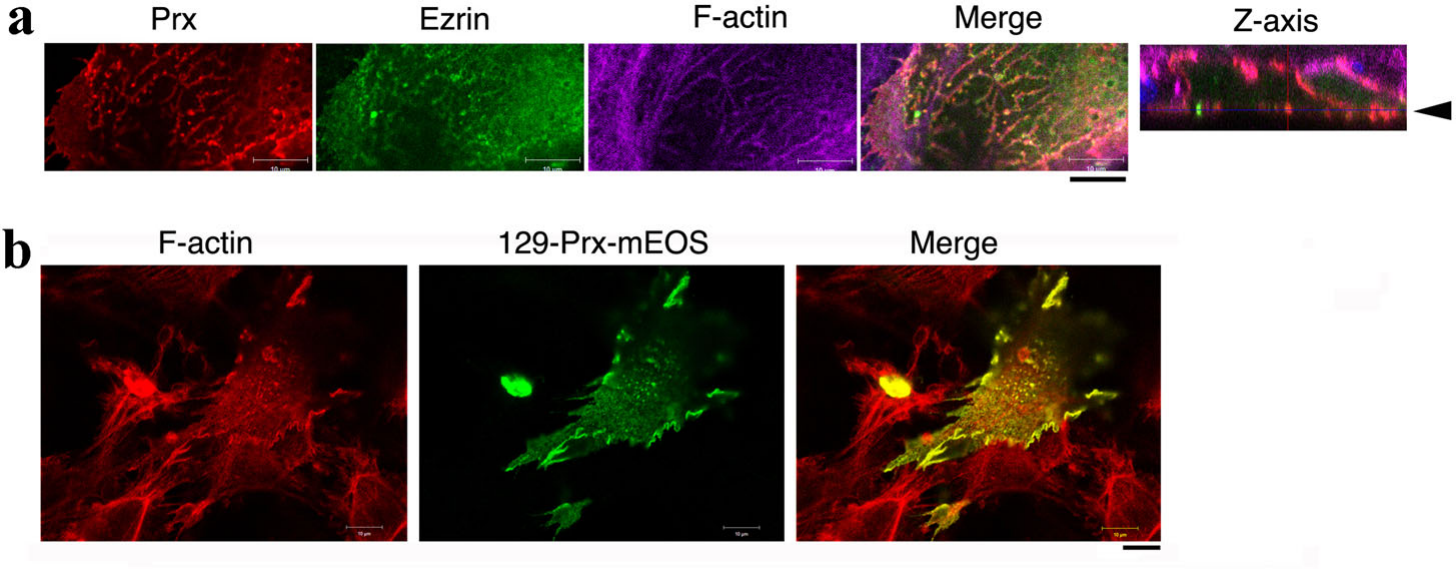
Distribution of periaxin, ezrin, and F-actin in primary lens culture cells in vitro. **a** Fluorescent confocal images show that 129-PRX proteins (red) colocalize with ezrin (green) and F-actin (purple), forming a distinct stripe-like network at the surface of differentiated fiber-like cells in vitro. These cells were derived from primary cultured lens epithelial cells of 129-WT mice and treated with 200 ng/ml bFGF in culture medium for 2 weeks. In the Z-axis image, the horizontal surface plane is indicated by an arrowhead. Scale bar: 10 μm. **b** Colocalization of 129-Prx-mEOS protein with F-actin in primary cultured lens epithelial cells transfected with 129-Prx-mEOS, co-labeled with rhodamine-phalloidin for F-actin. Primary cells were isolated from 129-WT mouse lenses. Scale bar: 10 μm.

Since PRX was originally identified as a component of the giant EPPD complex in lens fibers^50^, we investigated the distribution of 129-PRX at sites of F-actin and ezrin interaction in differentiated fiber cells in vitro. To induce lentoid differentiation, primary lens epithelial cells isolated from 129-WT lenses were cultured in high bFGF medium^53^. Immunostaining revealed that 129-PRX and ezrin colocalized with submembrane-associated F-actin networks, appearing as strip-like or dot-like structures in 129-WT-derived lentoid cells (Fig. 5a).

To further investigate the role of 129-PRX, we cloned 129-Prx using lens RNAs isolated from 129 mice and generated a 129-Prx-mEOS expression plasmid, in which 129-Prx was tagged at its C-terminal end with the mEOS fluorescent protein. This plasmid was transfected into primary cultured lens epithelial cells to evaluate the association between 129-PRX-mEOS and F-actin. Immunostaining revealed that 129-PRX-mEOS prominently accumulated at cell-cell contacts and cell surface protrusions/processes, where it colocalized with submembrane-associated actin filaments in transfected lens cells in vitro (Fig. 5b).

These findings suggest that 129-PRX acts as a gain-of-function scaffold protein, associating with cell surface protrusions, F-actin, and ezrin to regulate distinct membrane-cytoskeletal structures during fiber cell morphogenesis.

## Discussion

This study is the first to identify a specific gene variant that, across various mouse strain genetic backgrounds, contributes to increased cataract severity caused by another gene deficiency. Specifically, our findings demonstrate that age-related cataract formation results from the combined effects of a Cx46 gene deficiency and a particular Prx gene variant, with the severity being influenced by the underlying genetic backgrounds. We have mapped and characterized *Prx* gene variants between the 129 and B6 strain backgrounds, revealing that 129-PRX acts as a gain-of-function genetic modifier, enhancing cataract severity in Cx46KO mice. The gain-of-function 129-Prx variant is associated with significantly elevated and widely distributed PRX protein levels in lens fibers, which may be partly due to increased *Prx* gene transcription in the 129 strain background (Supplementary Fig. 1). These *Prx* gene variants affect the regulation of PRX-associated membrane-cytoskeletal structures, thus influencing the morphogenesis of inner fiber cells and modifying cataract formation caused by Cx46 deficiency in the lens. Previous studies have shown that age-related cataracts in Cx46KO lenses are associated with increased levels of water-insoluble and degraded crystallins^35,37^, activation of calpains^36,38,44^, elevated calcium levels, and calcium precipitates in the lens^39,41^. This study introduces a novel concept, suggesting that Cx46 deficiency-induced calcium elevation, combined with 129-PRX-mediated abnormal fiber membrane-cytoskeletal remodeling, contributes to the development of age-related nuclear cataracts.

This study reveals that both Cx46-mediated homeostasis and PRX-mediated fiber cell remodeling are intricately coupled to regulate lens cataract formation and transparency. Lens connexins Cx46 and Cx50 localize along both the short and long sides of hexagonally shaped fibers, as well as at ball-and-socket structures on the long sides, but are absent from the protrusions at the vertices^15,54,55^. Mutations in Cx46 and Cx50 are linked to various nuclear cataracts, which are associated with elevated calcium levels and calcium deposits in the lens^56,57^. Although calcium-dependent calpains play a critical role in lens fiber function and cataract formation^44,58,59^, there is no evidence to suggest that calpains act as genetic modifiers^46^.

This study demonstrates that 129-PRX acts as a gain-of-function genetic modifier, enhancing cataract severity in Cx46KO lenses. The *Prx* gene produces two protein isoforms through alternative splicing: a large-PRX (L-PRX) consisting of 1391 amino acids and a small-PRX (S-PRX) with 148 amino acids^60^. In this study, PRX specifically refers to L-PRX, which contains a PDZ-like domain and a basic domain with a nuclear localization signal in its N-terminal region, along with an extended C-terminal region that includes a PEVK-rich repeat domain and an acidic domain (Fig. 2a)^61^. PRX was initially identified as a key component in maintaining the myelin sheath in Schwann cells^60,62–65^. Its N-terminal PDZ domain facilitates PRX homodimerization or heterodimerization with S-PRX^66–68^, while the basic domain binds to the spectrin-like repeats of dystrophin-related protein 2 (Drp2), promoting the assembly of PRX/Drp2/Dag complexes that connect the extracellular matrix to the cytoskeletal network in Schwann cells^62,65,66^. However, PRX is the third most abundant myelin protein, comprising 16% of peripheral nervous system myelin protein, Drp2 accounts for only 0.2%^69,70^, indicating that PRX and Drp2 do not associate in a strict stoichiometric ratio within the PRX/Drp2/Dag complex. Moreover, the high prevalence of disease-associated mutations in the C-terminal region suggests the presence of other PRX associated complexes^71^. The C-terminal acidic domain interacts with the third cytoplasmic fibronectin-type III domain of integrin β4^72^, and truncation of the PRX C-terminal region reduces Drp2 complex formation^52^. This supports a two-pronged interaction model for PRX, in which the N-terminus binds to distinct membrane protein complexes such as Drp2/Dag, while the flexible C-terminal region serves as a dynamic linker, connecting to other protein complexes.

PRX is a multifunctional scaffold protein that likely utilizes a multi-pronged interaction strategy to regulate various protein complexes within membrane-cytoskeletal networks in lens fibers. Both wild-type B6 and 129 lenses are transparent, exhibiting typically hexagonal-shaped fibers and normal radial alignment. In contrast, periaxin knockout (PrxKO) lenses display irregular inner fiber cell morphology^48^, suggesting that endogenous B6-PRX and 129-PRX are sufficient to maintain proper fiber cell shape and architecture in wild-type lenses. However, it is specifically the presence of 129-PRX that is associated with the development of severe nuclear cataracts in Cx46KO lenses. Furthermore, variants of the human *Prx* gene (p.R129H, p.V1225M) have been linked to congenital cataract^73,74^, suggesting the potential role of PRX in cataractogenesis. Initially identified as a component of the EPPD complex, the specific function of PRX remained unclear^50^. The N-terminal basic domain of PRX interacts with the N-terminal F3 subdomain of ezrin in a “head-to-head and tail-to-tail” manner, suggesting that PRX may undergo conformational transitions between inactive and active states to modulate its interaction with ezrin^75^. Mass spectrometric analysis of PRX co-immunoprecipitated complexes revealed a variety of lens membrane and cytoskeletal proteins, including NrCAM, aquaporin-0 (Aqp0), Cx50, ankyrin B (AnkB), plectin-1, desmoyokin, ARVCF, catenin-α2, radixin, ezrin, spectrin (both α and β), filensin, phakinin, vimentin, and actin^48^. However, dystrophin-dystroglycan complexes were absent in lens fibers^48^. A subsequent study suggests that AnkB mediates the membrane tethering of PRX in lens fibers^51^. Conditional knockout of AnkB in mice leads to smaller lenses and nuclear cataracts^76^. In contrast, PrxKO mice develop normal-sized, transparent lenses but exhibit inner fiber cells with irregular sizes and misalignment^48^. The distinct phenotypic differences between PrxKO and AnkBKO mice suggest that the roles of PRX-AnkB-associated membrane-cytoskeletal protein complexes in fiber cell remodeling have not been fully elucidated and require further clarification^15,19,54^. The mechanism by which PRX interacts, either directly or indirectly, with AnkB, Aqp0, and the EPPD complex remains unclear^48^^,^^50^. Future investigation is needed to elucidate how PRX regulates inner fiber cell shape and size.

The 129-*Prx* and B6-*Prx* gene variants lead to the substitution of four amino acid residues–L234I, T431A, K439T and A1260V–within the extended C-terminal region of the PRX protein. These substitutions may influence the stability and turnover of PRX, possibly by altering its binding affinity with other proteins in the lens fibers. More studies are necessary to investigate the molecular mechanisms underlying the functional differences between 129-PRX and B6-PRX in lens fibers. Our recent mass spectrometry analysis of 129-PRX immunocomplexes confirms its association with several proteins, including ezrin, periplakin, desmoyokin, ankyrin, and Aqp0 (MP26) (unpublished data in collaboration with Dr. Kevin Schey, Vanderbilt University). 129-PRX likely interacts with ezrin- and AnkB-containing complexes, as well as other membrane-cytoskeletal networks, to regulate fiber cell morphogenesis and maintain structural integrity. We propose that the gain-of-function 129-PRX protein variant disrupts inner fiber cell remodeling, which, in combination with elevated calcium concentrations and calcium precipitation^39^, leads to irregular fiber cell shapes and sizes, as well as membrane/F-actin aggregates in 129-Cx46KO dense nuclear cataracts (Fig. 4d). In contrast, the absence of B6-PRX in inner fibers does not affect inner fiber cell remodeling in B6-Cx46KO lenses, which develop only mild nuclear cataracts. Additional studies are needed to identify proteins that interact with the highly flexible and intrinsically disordered C-terminal region^72^ of B6-PRX and 129-PRX, as supported by the predicted 3D PRX protein structure generated using SWISS-MODEL (https://swissmodel.expasy.org).

In summary, periaxin functions as a genetic modifier of nuclear cataract severity in Cx46KO mice. The combined effects of dysfunctional gap junctions in Cx46KO lenses and altered membrane-cytoskeletal protein complexes caused by the 129-*Prx* gene variant disrupt fiber cell architecture and lens homeostasis. These disruptions result in the aggregation and misregulation of membrane-cytoskeletal proteins and crystallins, increased calcium-dependent proteolysis, and calcium precipitation, all of which contribute to the development of dense nuclear cataracts in 129-Cx46KO lenses. Specifically, PRX-ezrin and PRX-AnkB-associated protein complexes may contribute to cataract severity in Cx46KO lenses by disrupting cytoskeleton-membrane integrity^14,17,25^. This study introduces a novel concept that membrane-cytoskeletal disorganization plays a critical role in cataract development. Our previous studies demonstrate that enhancing gap junction communication presents a promising strategy for cataract prevention^77,78^. Future cataract prevention approaches should prioritize improving intercellular communication and stabilizing cytoskeleton-membrane architecture to maintain lens transparency and function throughout aging.

## Methods

### Mouse breeding, genotyping and phenotyping

All mouse experimental procedures were approved by the Animal Care and Use Committee at University of California, Berkeley. Experiments were conducted following the National Institutes of Health guidelines for animal research. Wild-type and Cx46KO mice in the 129SvJae (129S4) and C57BL/6J strain backgrounds were used to generate intercrossed and backcrossed mice in 129/B6 mixed background for mapping genetic modifiers. Genotyping was performed with various linkage markers using mouse genomic DNAs. There are no noticeable differences in cataract severity between male and female Cx46KO mice at either 129 or B6 strain genetic backgrounds. Both male and female mice were used to minimize mouse usage numbers in this study.

A standard PCR method was used to genotype different satellite markers. The genotyping of the Cx46KO allele was assessed by a PCR method previously reported^35^. The following procedure was used to prepare the genomic DNAs: a tiny piece of tail tissue was collected and digested in a lysis buffer (100 mM Tris pH 8.0, 5 mM EDTA, 0.2% SDS and 200 mM NaCl) with proteinase K at 55 °C, then an equal amount of isopropanol was added to precipitate DNA, and the DNA pellet was rinsed with 70% ethanol and dissolved in TE buffer (10 mM Tris-Cl pH 7.5 and 1 mM EDTA). Two pools of 129/B6 mixed background tail DNA samples with severe and mild cataracts were prepared and subjected to the QTL mapping analysis as described previously^79,80^. A list of linkage markers was obtained from a public database (http://www.informatics.jax.org/marker) and used for mapping genetic modifiers by using mouse genomic DNA samples isolated from different generations of 129/B6 Cx46KO mice backcrossed with 129-Cx46KO mice (see details in the “results”).

Mouse lens phenotyping was assessed through visual examination of cataracts in live mice using slit lamp examination. Mouse pupil was dilated with a drop of 1:1 mixture of 1% Atropine Sulfate and 2.5% Phenylephrine Hydrochloride ophthalmic solution, followed by slit lamp examination to evaluate the cataract severity.

### Imaging and light scattering quantification of mouse lenses

Fresh lenses, immediately dissected from euthanized mice, were immersed in PBS for imaging under a Leica MZ16 dissecting scope with a digital camera. Light scattering of clear and cataract lenses was measured using the HR 2000CG_UV_NIR high resolution spectrometer and a QP400-2-UV-VIS fiber optic cable (400 μm cable diameter) (Ocean Optics, Dunedin, FL) as described previously^81^. In brief, lenses were illuminated by a white light source perpendicular to the lens equator, and scattered light was captured by the optic fiber with a whole acceptance angle of 24.8°. Spectrums were recorded and saved for later comparison. Each lens was measured twice in succession to show repeatability. The measurement data were represented as graphs with wavelength on the x-axis and light intensity on the y-axis. Dense cataracts in *129-Cx46KO* lenses had more scattered light, showing increased light scattering intensity measured by the detector. Measurements were stored as ASCII files, and the Matlab program was used for calculating the area under the curve. For each genotype, six lenses from three different mice were used to determine the average light scattering intensity. Normalized intensity was calculated by subtracting the under-curve area of wild-type lenses, which are transparent. The average normalized intensity and standard error were plotted in Excel. Statistical significance was determined using the student’s *t*-test.

### Morphological characterization of lens sections and single fibers

For immunohistology study, mouse lenses were fixed in 4% paraformaldehyde (PFA) in phosphate buffered saline (PBS) at room temperature for 30 min, the fixed lenses were then washed 3 times with PBS before cutting into 150 μm-thick sections in cross- and anterior-posterior (AP) orientations with a vibratome (Leica VT1000 S). The sections were post fixed for 2 min in 4% PFA in PBS then washed 3 times with PBS, and were incubated with blocking solution (3% w/v bovine serum albumin, 3% v/v normal goat serum, 0.3% v/v Triton X-100 in PBS) for 2 h, followed by incubation with specific antibodies in blocking buffer for overnight at 4 °C. After primary antibody incubation, the sections were washed with PBS then incubated for overnight at 4 °C or 3 h at 37 °C with fluorescent labeled secondary antibodies, rhodamine-wheat germ agglutinin (WGA) (Vector Laboratories) and FITC-phalloidin (Thermo Fisher). Samples were finally washed in PBS and mounted with Vectashield antifade mounting medium with DAPI (Vector Laboratories). Either single or Z-stack images were collected with a confocal microscope (LSM 700, Zeiss). The following antibodies were used: rabbit anti-periaxin (Sigma-Aldrich HPA001868) and rabbit anti-ezrin (#3145, Cell Signaling).

Individual fibers were isolated from 200 to 300 μm thick AP lens sections, which were carefully cut using a blunt blade. The surface of the AP sections was gently teased apart with fine tweezers to release individual elongated fibers from the middle regions, which were then subjected to fluorescent labeling. Single fibers were selected from both the peripheral and interior regions of fluorescent-labeled lens AP sections, based on their distance from the lens capsule. The acquired images of peripheral differentiated fibers and interior MF enable the monitoring of changes in fiber cell morphology and surface interlocking structures at different locations within the lens.

### Western blotting analysis

Lenses were weighed and homogenized in 0.1 M NaCl, 50 mM Na_2_HPO_4_ buffer, using a ratio of 10 mg lens weight per 500 μl buffer. After centrifugation at 14,000 rpm for 15 min, the insoluble pellet was collected and washed twice with the buffer. Soluble protein concentration was measured, and 50 μg of protein was loaded per sample lane for gel separation. The insoluble pellets were resuspended in sample buffer (60 mM Tris pH 6.8, 2% SDS, 10% glycerol, and 0.001% bromphenol blue, and 10 mM DTT) at a ratio of 100 μl of sample buffer per lens. About 40 μl of the insoluble sample was loaded onto a 4–12% NuPAGE Bis-Tris Gel (Invitrogen, Carlsbad, CA) for protein separation. The gel was then transferred to a PVDF membrane (Bio-Rad Laboratories) using a standard method. Rabbit anti-periaxin antibody (Sigma-Aldrich HPA001868) and monoclonal anti-β-actin antibody (A5441, Sigma-Aldrich) were used. The SuperSignal West Pico Chemiluminescent substrate kit (Thermo Scientific, Rockford, IL) was used to develop the western blot, and images were captured by the Azure Biosystems c600. The Western blot results were repeated using at least three independent sets of lens samples from different mice.

### Quantitative real-time PCR (qRT-PCR) analysis

Total RNA was extracted from lenses of B6-WT, 129-WT, B6-Cx46KO and 129-Cx46KO mice using TRIzol™ reagent (Invitrogen, Carlsbad, CA). Four lenses (from two mice) were pooled for each genotype. The cDNAs were synthesized using the Super-Script II™ first strand cDNA synthesis system (Invitrogen) according to the manufacturer’s protocol. qRT-PCR was performed using a primer-probe set targeting a 144 bp region spanning exons 4 to 5 of the *Prx* gene. The sequences were: forward primer, 5′- AGC TAT GGA GGC CAG GAG-3′; reverse primer, 5′- AGC TCA CGG ACA AAG ATT CC-3′; and probe, 5′-FAM- CTG AGA CGG GCG GAG TTG GT-BHQ-3′. GAPDH was used as an internal control, with the following primer-probe set: forward primer, 5′-GCC CAT CAC CAT CTT CCA G-3′; reverse primer, 5′-TTT GGC TCC ACC CTT CAA G-3′; and probe, 5′-FAM-ACA CCA GTA GAC TCC ACG ACA TAC TCA G-3′. Each cDNA sample was run in quadruplicate. Absolute gene copy numbers were calculated using a standard curve generated from serial dilutions of a pcDNA3-129Prx plasmid. qRT-PCR reactions were performed on a Realplex qPCR cycler (Eppendorf) using the following program: 95 °C for 10 min, followed by 40 cycles of 95 °C for 15 s and 57.5 °C for 2 min. Data were analyzed using the equipped software (Eppendorf). Each bar in the resulting graph represents the average *Prx* copy number normalized to the average GAPDH copy number from quadruplicated reactions. Data are represented as mean ± SD.

### Generation of Prx cDNAs and pmEos2 tagged-Prx plasmids

Both 129-Prx cDNA and B6-Prx cDNA (4.2 kb), covering the coding sequence, were cloned into the Zero Blunt TOPO vector (Invitrogen). The genetic variants of these constructs were confirmed by DNA sequencing of RT-PCR products derived from total RNAs isolated from 129 and B6 lenses. The following primer pair was used for PCR amplification: Prx5′-GACTCTCTGCAGAGCTATGGAGGCCAGGAG and Prx3′-TTCAGATGGCAGCAGCCTG. The pmEos2-N1-129-Prx plasmid was generated by tagging mEos2 in-frame at the C-terminal end of Prx. The full length 129-Prx cDNA was first digested with EcoRI from the cloned TOPO vectors and subcloned into the pmEos2-N1 vector (CMV promoter) at EcoRI site, located just upstream of the mEos2 gene. Plasmids with the correct orientation were selected to ensure the mEOS2 tag was properly inserted at the C-terminal end of *Prx*. DNA sequencing confirmed that the *Prx* stop codon and multiple cloning sites between *Prx* and mEos2 were replaced with a linker sequence GGGGATCCACCGGTCGCCACC, allowing the C-terminal end of *Prx* to be in-frame with the N-terminal end of mEos2 protein through a peptide liker GDPPVAT.

### Lens primary epithelial cell culture, differentiation into fiber-like cells, and plasmid transfection

Primary lens epithelial cell culture: 129-WT mouse lenses were dissected and incubated in 0.05% trypsin-EDTA (Gibco, cat#: 25300-054) at 37 °C for 10 min, after which any remaining tissue on the lens surface was removed. The lens capsules were carefully peeled off using forceps and transferred to a sterile tube containing 100 µl of dispase in Advanced DMEM-F12 medium (2 U/ml, Sigma, cat#: D4693). Following 5 min of dispase treatment, 100 µl of 10 × TrypLE (Gibco, cat#: A12177-02) was added and incubated for 10 min. The cell suspension was then centrifuged at 1000 rpm for 4 min. The cell pellet was resuspended in culture medium and seeded onto culture dishes. The culture medium consisted of Advanced DMEM/F12 (Gibco, cat#: 12634-010), supplemented with 1x penicillin-streptomycin (Gibco, cat#: 15140-122), 1x GlutaMax (Gibco, cat#: 35050-061), 2% fetal bovine serum (FBS, Gibco, cat#: 26140-079), 1x B-27 (Gibco, cat#: 17504-044), and 5 µM SB431542 (Stemgent, cat# 04-0010-10, 20 mM stock prepared in DMSO). The culture medium was changed every other day. Within 7–10 days, epithelial cells isolated from a lens would proliferate to several million cells.

Differentiation into fiber-like cells: The cultured lens epithelial cells, upon reaching over 50% confluency, were treated with freshly prepared culture medium containing 200 ng/ml bFGF for at least 2 weeks to induce differentiation into fiber-like cells in vitro. The differentiated cells were then fixed for immunostaining analysis.

Plasmid transfection: First-generation lens epithelial cells were passaged once and cultured for an additional 3 days before being transfected with pmEOS-N1/B6-Prx and pmEOS-N1/129-Prx plasmids via electroporation using a Bio-Rad Gene Pulser MXcell™ ShockPod™ Cuvette Chamber. For electroporation, ~10^6^ cells in 0.8 ml PBS were mixed with 8 μg of plasmid DNA in 0.4 cm cuvettes. Electroporation was performed following the manufacturer’s protocol. The electroporated cells were then seeded into two 35-mm glass-bottom culture dishes. Transfected cells were examined directly for mEos fluorescent protein expression using a confocal microscope and fixed for immunostaining analysis after 48 h.

### Reporting summary

Further information on research design is available in the Nature Portfolio Reporting Summary linked to this article.

## Supplementary information

Supplementary Information

Description of Additional Supplementary Files

Supplementary Data

Reporting Summary

## Data Availability

All data supporting the findings of this study are included in the paper and its Supplementary Information. Source data for figures are provided in Supplementary Data. Additional information is available from the corresponding author upon reasonable request.
