# Supplementary material for: Periaxin gene variants are linked to age-related cataracts in Cx46 deficient lenses
Source: Commun Biol. 2025 Sep 24;8:1356. doi: 10.1038/s42003-025-08722-4 (PMC12460658; doi:10.1038/s42003-025-08722-4)
Supplement: Supplementary file 1 — Supplementary Information [file 42003_2025_8722_MOESM1_ESM.pdf]

Supplementary Information

Periaxin gene variants are linked to age-related cataracts in Cx46 deficient lenses

Chun-hong Xia[1], Eddie Wang[1], Lin Li[1], Dong Wang[1], Bo Chang[2], Mei Li[1], and Xiaohua Gong[1,3*]

1. Vision Science and School of Optometry, University of California, Berkeley, Berkeley, CA, 94720 USA
2. The Jackson Laboratory, Bar Harbor, ME, 04609 USA
3. The UCB/UCSF jointed graduate program in Bioengineering, University of California, Berkeley, Berkeley, CA, 94720 USA

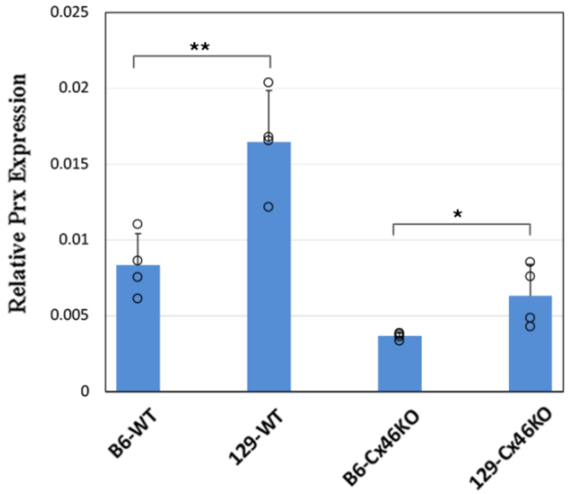

Supplementary Figure 1. Quantitative comparison of *Prx* transcription levels in lenses of B6-WT, 129-WT, B6-Cx46KO, and 129-Cx46KO mice using quantitative real-time PCR (qRT-PCR). The 129-WT lenses showed approximately a twofold increase in *Prx* transcript levels compared to B6-WT lenses, and 129-Cx46KO lenses exhibited a similar twofold increase compared to B6-Cx46KO lenses. WT lenses were isolated from two-week-old mice, while Cx46KO lenses were collected from three-week-old mice. The bar graph represents *Prx* expression levels normalized to *GAPDH,* based on quadruplicated reactions. Data are represented as mean ± SD, with statistical analysis performed using Student's *t*-test (*$P < 0.05$, **$P < 0.01$; n = 4).

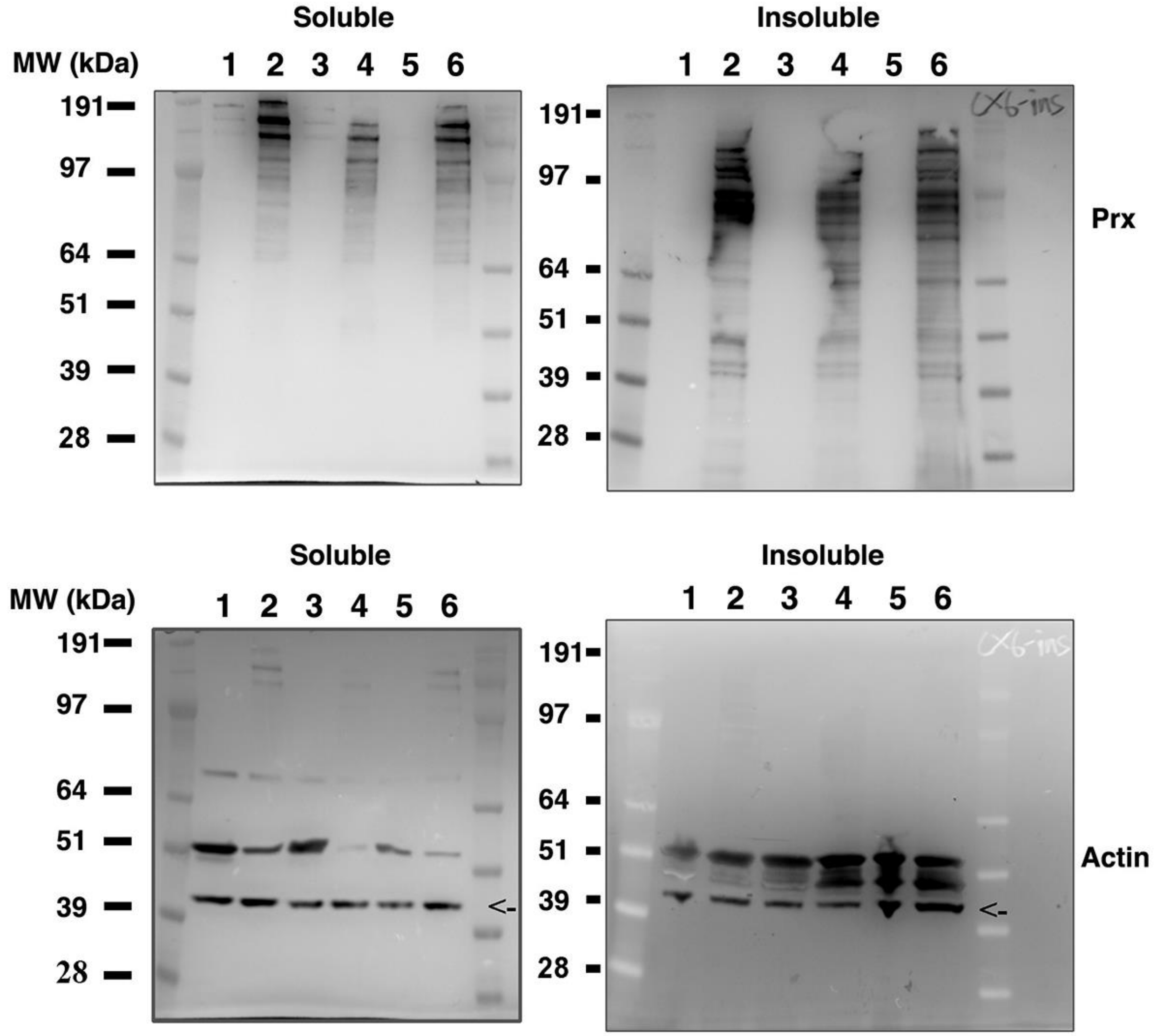

Supplementary Figure 2. Raw Western blot data corresponding to Figure 2. Water-soluble and water-insoluble protein fractions from 3-week-old mouse lenses were subjected to Western blotting. The lanes are labeled as follows: (1) B6 wild-type (B6-WT), (2) 129 wild-type (129-WT), (3) B6-*Cx46KO,* (4) 129-*Cx46KO,* (5) *B6L/B6L-Cx46KO*, and (6) *B6S/B6S-Cx46KO*. The upper panels show images of membranes immunoblotted with anti-periaxin (PRX) antibody. The lower panels show images of membranes probed with anti-β-actin antibody. Arrows indicate β-actin bands migrating above 39 kDa; additional bands near 51 kDa are from another antibody.
