# Supplementary material for: Periaxin gene variants are linked to age-related cataracts in Cx46 deficient lenses
Source: Commun Biol. 2025 Sep 24;8:1356. doi: 10.1038/s42003-025-08722-4 (PMC12460658; doi:10.1038/s42003-025-08722-4)
Supplement: Supplementary file 2 — Description of Additional Supplementary Files [file 42003_2025_8722_MOESM2_ESM.pdf]

Description of Additional Supplementary Files

File name- Supplementary Data

File description - Source data for figures are provided in Supplementary Data
